# Risk factors and recurrence of cause-specific postpartum hemorrhage: A population-based study

**DOI:** 10.1371/journal.pone.0275879

**Published:** 2022-10-14

**Authors:** Lorentz Erland Linde, Svein Rasmussen, Dag Moster, Jörg Kessler, Elham Baghestan, Mika Gissler, Cathrine Ebbing

**Affiliations:** 1 Department of Clinical Science, University of Bergen, Bergen, Norway; 2 Department of Global Public Health and Primary Care, University of Bergen, Bergen, Norway; 3 Department of Pediatrics, Haukeland University Hospital, Bergen, Norway; 4 Department of Obstetrics and Gynaecology, Haukeland University Hospital, Bergen, Norway; 5 Finnish Institute for Health and Welfare, Department of Knowledge Brokers, Helsinki, Finland; 6 Karolinska Institute, Department of Molecular Medicine and Surgery, Stockholm, Sweden; University of Oslo, NORWAY

## Abstract

**Objective:**

To explore risk profiles of the different types of postpartum hemorrhage (PPH >500ml or severe PPH >1500ml) and their recurrence risks in a subsequent delivery.

**Methods:**

With data from The Medical Birth Registry of Norway and Statistics Norway we performed a population-based cohort study including all singleton deliveries in Norway from 1967–2017. Multilevel logistic regression was used to calculate odds ratio (OR), with 95% confidence interval (CI), with different PPH types (PPH >500ml or PPH >1500ml (severe PPH) combined with retained placenta, uterine atony, obstetric trauma, dystocia, or undefined cause) as outcomes.

**Result:**

We identified 277 746 PPH cases of a total of 3 003 025 births (9.3%) from 1967 to 2017. Retained placenta (and/or membranes) was most often registered as severe PPH (29.3%). Maternal, fetal, and obstetric characteristics showed different associations with the PPH types. Male sex of the neonate was associated with reduced risk of PPH. This effect was strongest on PPH due to retained placenta (adjusted OR, (aOR): 0.80, 95% CI 0.78–0.82), atony (aOR 0.92, 95% CI: 0.90–0.93) and PPH with undefined cause (aOR 0.96, 95% CI: 0.95–0.97). Previous cesarean section showed a strong association with PPH due to dystocia (aOR of 13.2, 95% CI: 12.5–13.9). Recurrence risks were highest for the same type: PPH associated with dystocia (aOR: 6.8, 95% CI: 6.3–7.4), retained placenta and/or membranes (aOR: 5.9, 95% CI: 5.5–6.4), atony (aOR: 4.0, 95% CI: 3.8–4.2), obstetric trauma (aOR: 3.9, 95% CI: 3.5–4.3) and PPH of undefined cause (aOR: 2.2, 95% CI: 2.1–2.3).

**Conclusion:**

Maternal, fetal and obstetric characteristics had differential effects on types of PPH. Recurrence differed considerably between PPH types. Retained placenta was most frequently registered with severe PPH, and showed strongest effect of sex; delivery of a boy was associated with lower risk of PPH. Previous cesarean increased the risk of PPH due to dystocia.

## Introduction

Postpartum hemorrhage (PPH) is the leading direct cause of maternal mortality worldwide [1]. Main types of PPH described in literature are PPH associated with uterine atony and retention of the placenta [2–5]. It is important to disentangle the different types of PPH, in order to gain insight into the pathophysiological mechanisms, and to find potential clinical interventions that may reduce occurrence and severity of PPH.

In studies on risk factors of types of PPH, emphasis has usually been placed on two main causes of PPH; uterine atony [4,6,7] or retained placenta [3,8–11], while important types, like PPH caused by obstetric trauma or dystocia, are widely ignored. Further, the considerable variation in estimated occurrence rates between populations [2,3,12], exceeds what could be expected to be caused by environmental and genetic variations.

Studies have reported associations of PPH (in general) with demographic [3,10,13–17], and pregnancy-related factors [3,10], induction of labor [11], obstetric history, including recurrence risk [9–11], and complications related to the fetus, placenta, membranes and umbilical cord [9,10,18–21], while studies on risk factors of type specific PPH are scarce. Thus, we aimed to explore risk profiles of different PPH types through our specific objectives: to calculate the effects of demographic and pregnancy-related factors, obstetric history and complications related to the fetus, placenta, membranes and umbilical cord, and to investigate the recurrence risk of the different types of PPH in the Norwegian population.

## Material and methods

### Data sources

The Medical Birth Registry of Norway (MBRN), established in 1967, is a mandatory register containing information of all births in Norway [22]. For our main analyses we identified singleton births in the MBRN from 1967 to 2017 with gestational age at birth of ≥22 weeks and spontaneous onset or induction of labor. This selection excluded planned cesarean sections, but included cesareans after onset or induction of labor. Gestational age was estimated from the last menstrual period and based on ultrasonography when data for the last menstrual period were lacking. Information on the parental education level and country of birth was provided by Statistics Norway and linked with the birth registry using the unique national identification number of each parent.

### Record linkage

During the period from 1967 to 2017, 3 003 025 births were registered. Using the national identification number, we linked the first two births in women who gave their first birth in 1967 or later, to assess the risk of PPH types according to pregnancy- and birth-related factors and obstetric history, including recurrence risk.

### Ethics statement/approval

The study was approved by the Regional Committee for Medical and Health Research Ethics (2013/1484) and the registry owners (the Medical Birth Registry of Norway, the Norwegian Institute of Public Health, Statistics Norway and the Norwegian Tax Administration).

### Outcome variables

The main outcome variables were PPH defined as the loss of more than 500ml of blood during labor or within 24 hours postpartum (hereafter referred to as PPH) in combination with one of seven predefined types of PPH described below. The PPH types were not mutually exclusive as more than one PPH type could be recorded in the same delivery.

Before 1999 bleeding volume during labor was registered to MBRN as free text and categorized as PPH if the volume was more than 500ml. In 1999, the notification form was upgraded with new, predominantly categorical, variables: PPH 500ml to 1500ml: Blood loss from 500ml to 1500ml during labor or within the first 24 hours after labor; and PPH >1500 ml: blood loss of more than 1500ml during or within 24 hours after labor or the need for blood transfusion (regardless of bleeding volume) (hereafter referred to as severe PPH) [22–24].

PPH types were defined as PPH combined with each of the following complications:

#### 1 Retained placenta and/or membranes

Defined as lack of expulsion of the placenta within 30 minutes of delivery [25], or retention of membranes. This was notified to the MBRN by plain text before 1999 and by check box from 1999, or by plain text as manual removal of the placenta, postpartum uterine curettage or abnormally invasive placenta from 1967 to 2017.

#### 2 Uterine atony

Failure of the uterus to contract adequately following delivery [26], notified in the MBRN by plain text before 1999 and by check box from 1999.

#### 3 Obstetric trauma

Notified in the MBRN as perineal laceration (1^st^ to 4^th^ degree) (by plain text before 1999 and by check boxes from 1999) or notified by plain text as other obstetric trauma (e.g., cervical or vaginal trauma) or inversio uteri from 1967.

#### 4 Dystocia

Duration of labor with spontaneous onset extends beyond the normal duration defined by the World Health Organization, (based on observational studies from 1973–2018) [27]. First stage (time from five centimeters to full cervical dilatation) 12 and 10 hours in first and subsequent labors, respectively. Second stage (time from full cervical dilatation to birth) three and two hours in first and subsequent labors, respectively. Protracted labor or cephalopelvic disproportion has been notified in the MBRN by plain text before 1999 and from 1999 by check box.

#### 5 Undefined PPH cause

PPH without recorded cause.

#### 6 Placental abruption

Notified in the MBRN before 1999 by plain text, and from 1999 by check box.

#### 7 Placenta previa

Notified in the MBRN before 1999 by plain text, and from 1999 by check box.

### Independent variables

Independent variables were demographic characteristics (maternal age, country of origin, marital status, education), obstetric history, pregnancy and fetal complications, and characteristics of the placenta, membranes, or umbilical cord. Independent variables also included a history of PPH (including the type of PPH) in the first delivery, inter-delivery interval, change of father between pregnancies, and previous cesarean section. Our analyses included possible confounding factors: maternal age (in five categories), parity, marital status, inter-delivery interval, mother’s country of birth, level of education, and the period of birth divided into five groups of approximately equal length (1967–1977, 1978–1987, 1988–1997, 1998–2007 and 2008–2017). (S1 File) includes additional details.

### Statistical analysis

We used multilevel logistic regression analyses to calculate odds ratios (ORs) with 95% confidence intervals (CIs) for PPH types as outcomes, and variables related to demographic characteristics, obstetric history, pregnancy, and fetal complications, and characteristics of the placenta, membranes, and umbilical cord as exposures. We also calculated ORs for PPH types in the actual birth as the outcomes and previous PPH types as exposure variables.

We used sensitivity analyses to assess if the associations studied persisted after adjusting for unmeasured confounders and to indicate potentially false positive associations by chance conducting multiple analyses. (S1 File) includes additional details.

The statistical analyses were performed using SPSS (version 25) and MLwiN (version 3.05).

## Results

**Fig 1:**
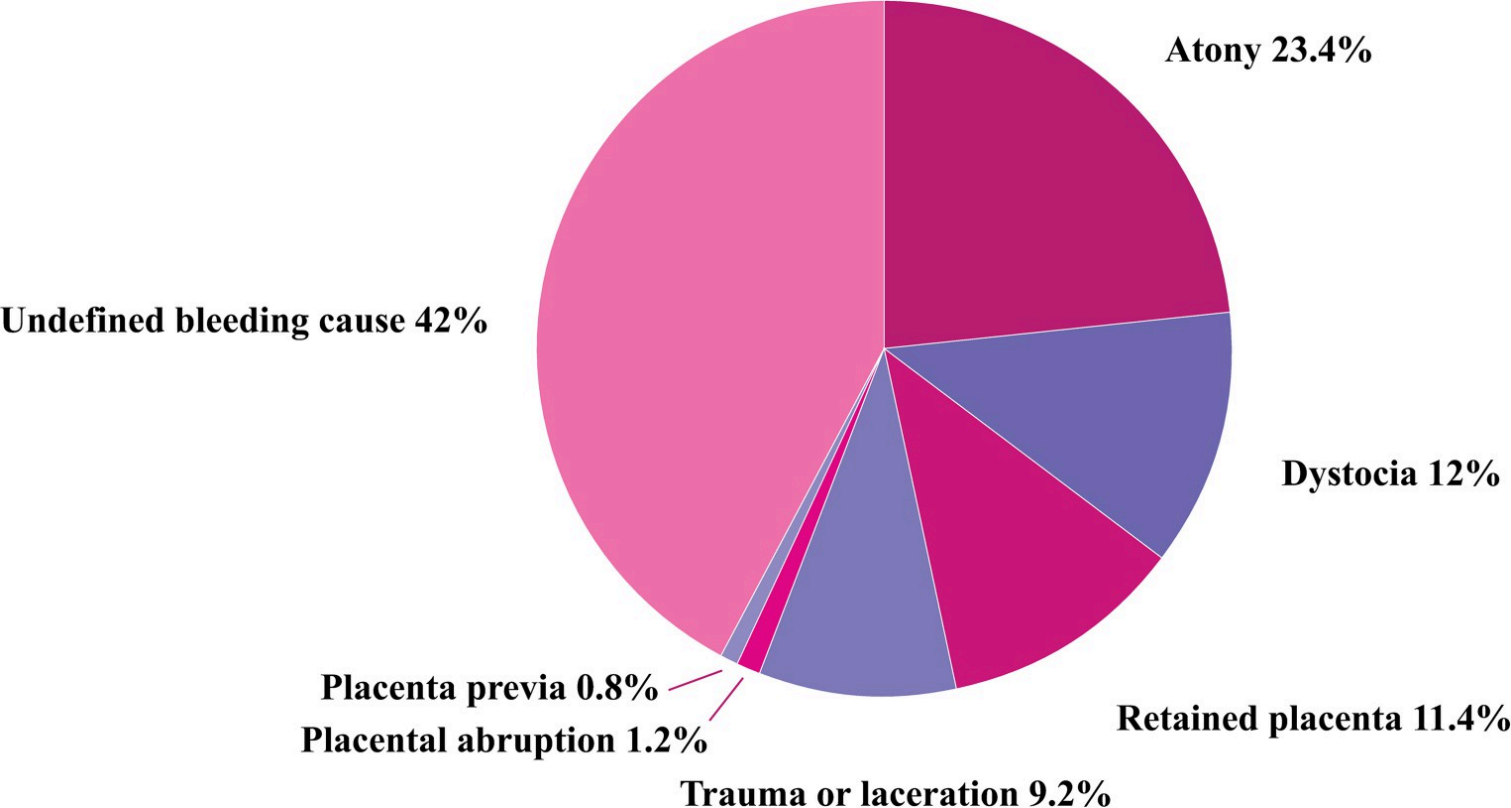
Occurrence of type specific postpartum hemorrhage (>500ml) (1967–2017); singleton births, ≥22 weeks of gestation.

Table 1 and Fig 1 shows occurrence of type specific PPH among singleton pregnancies with gestational age at birth of ≥22 weeks of gestation. The distribution of PPH types, in decreasing order of group size, included 42.0% (n = 131 170) without specified cause of PPH, 23.4% (n = 73 284) due to atony, 12.0% (n = 37 597) dystocia, 11.4% (n = 35 664) retained placenta and/or membranes, and 9.2% (n = 28 673) obstetric trauma. Placental abruption and placenta previa were registered as cause of PPH in 1.2% (n = 3598) and 0.8% (n = 2542), respectively. The total number of PPH registrations (n = 312 528) exceeded the total number of births with PPH (n = 277 746), since more than one PPH type could be recorded in the same birth.

**Table 1:** Occurrence of type specific postpartum hemorrhage (PPH); singleton births ≥22 weeks of gestation.

		All types			Retained placenta			Atony			Trauma or laceration			Placental abruption			Placenta previa			Dystocia			Undefined bleeding cause		
		(n)	%^a^	%^b^	(n)	%^a^	%^b^	(n)	%^a^	%^b^	(n)	%^a^	%^b^	(n)	%^a^	%^b^	(n)	%^a^	%^b^	(n)	%^a^	%^b^	(n)	%^a^	%^b^
**All PPH (>500ml)**	1967–2017	312528	100.0		35664	11.4		73284	23.4		28673	9.2		3598	1.2		2542	0.8		37597	12.0		131170	42.0	
**Mild PPH (500–1500ml)**	1999–2017	191730	100.0	87.2	17455	9.1	70.7	39048	20.4	84.3	16760	8.7	86.6	1785	0.9	77.9	1647	0.9	76.1	26644	13.9	87.0	88391	46.1	93.6
**Severe PPH (>1500ml)**	1999–2017	28149	100.0	12.8	7229	25.7	29.3	7276	25.8	15.7	2586	9.2	13.4	506	1.8	22.1	517	1.8	23.9	3980	14.1	13.0	6055	21.5	6.4

^a^ Distribution between types in percent (row percent)

^b^ Proportions according to severity of PPH types (column percent) (since 1999, when severe PPH was specified).

Severe PPH (registered after 1999, 28 149 type specific cases) showed a different distribution with 25.8% (n = 7276) caused by atony and 25.7% (n = 7229) by retained placenta, followed in decreasing order: undefined bleeding cause 21.5% (n = 6055), dystocia 14.1% (n = 3980), obstetric trauma 9.2% (n = 2586), placenta previa 1.8% (n = 517) and placental abruption 1.8% (n = 503) (Table 1).

**Fig 2:**
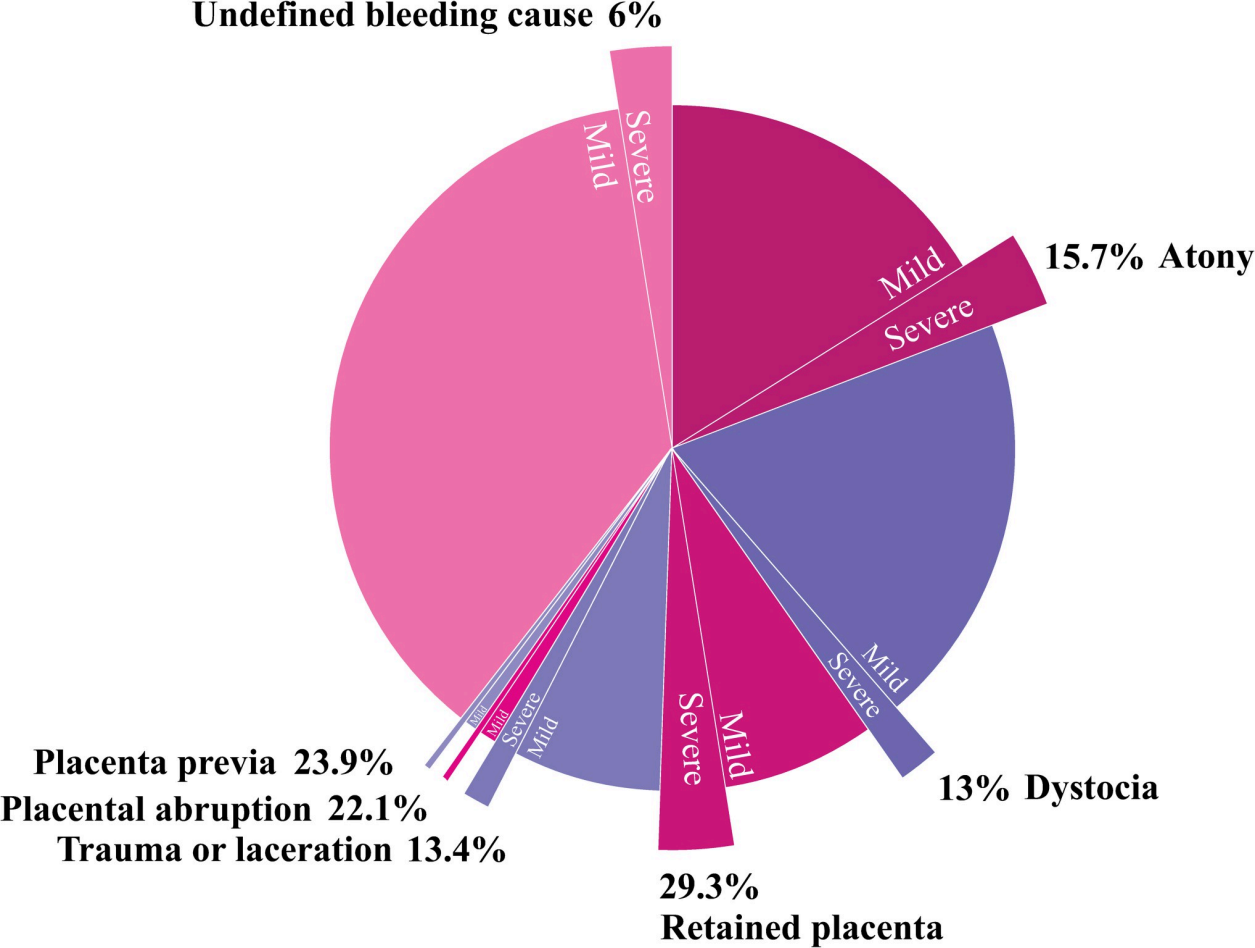
Proportions of severe postpartum hemorrhage (>1500ml) within type specific postpartum hemorrhage (1999–2017); singleton births, ≥22 weeks of gestation.

Women who had PPH caused by retained placenta were more often registered with severe PPH (29.3%) compared with other categories of PPH (Table 1, Fig 2), while only 6.4% of those with undefined cause of PPH were severe PPH cases.

Table 2 shows the distribution of maternal, pregnancy and birth characteristics in types of PPH.

**Table 2:** Distribution of maternal, pregnancy and birth characteristics in types of postpartum hemorrhage (PPH >500ml); singleton births, ≥22 weeks of gestation.

		All types		Retained placenta		Atony		Trauma or laceration		Placental abruption		Placenta previa		Dystocia		Undefined bleeding cause	
		(n)	%	(n)	%	(n)	%	(n)	%	(n)	%	(n)	%	(n)	%	(n)	%
**Maternal age (years)**	<20	9332	3.0	863	2.4	2523	3.4	1054	3.7	106	2.9	13	0.5	801	2.1	3972	3.0
	20–24	55452	17.7	5711	16.0	14388	19.6	6045	21.1	576	16.0	136	5.4	5829	15.5	22767	17.4
	25–29	103710	33.2	11198	31.4	25115	34.3	10641	37.1	1098	30.5	513	20.2	13315	35.4	41830	31.9
	30–34	92251	29.5	11223	31.5	20744	28.3	7685	26.8	1086	30.2	922	36.4	11806	31.4	38785	29.6
	35–39	42678	13.7	5494	15.4	8802	12.0	2807	9.8	578	16.1	746	29.3	4914	13.1	19337	14.7
	40–44	8595	2.8	1112	3.1	1621	2.2	414	1.4	148	4.1	200	7.9	884	2.4	4216	3.2
	≥45	510	0.2	63	0.2	91	0.1	27	0.1	6	0.2	12	0.5	48	0.1	263	0.2
**Parity**	0	157617	50.4	16033	45.0	34849	47.6	18661	65.1	1290	35.9	739	29.1	28690	76.3	57355	43.7
	1	99371	31.8	12316	34.5	24554	33.5	7485	26.1	1217	33.8	1032	40.6	6838	18.2	45929	35.0
	2	38856	12.4	5204	14.6	9792	13.4	1931	6.7	668	18.6	469	18.5	1511	4.0	19281	14.7
	3	10994	3.5	1421	4.0	2731	3.7	416	1.5	240	6.7	203	8.0	372	1.0	5611	4.3
	4	3983	1.3	463	1.3	928	1.3	140	0.5	128	3.6	71	2.8	135	0.4	2118	1.6
	≥5	1707	0.5	227	0.6	430	0.6	40	0.1	55	1.5	28	1.1	51	0.1	876	0.7
**Year of delivery**	1967–1969	9042	2.9	779	2.2	2268	3.1	430	1.5	155	4.3	36	1.4	182	0.5	5192	4.0
	1970–1979	24384	7.8	2718	7.6	6836	9.3	1701	5.9	318	8.8	61	2.4	853	2.3	11897	9.1
	1980–1989	25760	8.2	3510	9.8	8007	10.9	3108	10.8	384	10.7	99	3.9	2157	5.7	8495	6.5
	1990–1999	40825	13.1	4990	14.0	11430	15.6	4559	15.9	543	15.1	230	9.0	4669	12.4	14404	11.0
	2000–2009	96120	30.8	12294	34.5	22990	31.4	7858	27.4	1179	32.8	1003	39.5	12572	33.4	38224	29.1
	2010–2017	116397	37.2	11373	31.9	21753	29.7	11017	38.4	1019	28.3	1113	43.8	17164	45.7	52958	40.4
**Previous 1st trimester spontaneous abortion** ^**a**^	No	177174	80.4	19011	76.8	37367	80.5	16185	83.6	1759	76.5	1570	72.5	25314	82.5	75968	80.3
	Yes	43142	19.6	5737	23.2	9046	19.5	3181	16.4	539	23.5	597	27.5	5362	17.5	18680	19.7
**1st trimester bleeding**	**No**	303194	97.0	33940	95.2	71180	97.1	28130	98.1	3473	96.5	2386	93.9	36477	97.0	127608	97.3
	**Yes**	9334	3.0	1724	4.8	2104	2.9	543	1.9	125	3.5	156	6.1	1120	3.0	3562	2.7
**Previous cesarean section**	No	279188	89.3	32597	91.4	68194	93.1	25881	90.3	3119	86.7	2050	80.6	33216	88.3	114131	87.0
	Yes	33340	10.7	3067	8.6	5090	6.9	2792	9.7	479	13.3	492	19.4	4381	11.7	17039	13.0
**Preeclampsia**	No	299179	95.7	34182	95.8	70433	96.1	27615	96.3	3310	92.0	2502	98.4	35943	95.6	125194	95.4
	Yes	13349	4.3	1482	4.2	2851	3.9	1058	3.7	288	8.0	40	1.6	1654	4.4	5976	4.6
**Smoking in start of pregnancy** ^**a**^	No	165227	89.1	18531	86.7	36750	89.6	15846	91.1	1491	79.1	158	43.3	23531	90.3	68920	89.2
	Occasionally	2880	1.6	333	1.6	604	1.5	258	1.5	52	2.8	28	7.7	405	1.6	1200	1.6
	Yes	17275	9.3	2509	11.7	3680	9.0	1288	7.4	341	18.1	179	49.0	2117	8.1	7161	9.3
**Gestational or pregestational diabetes mellitus**	No	302981	96.9	34768	97.5	71523	97.6	28001	97.7	3510	97.6	2459	96.7	36002	95.8	126718	96.6
	Yes	9547	3.1	896	2.5	1761	2.4	672	2.3	88	2.4	83	3.3	1595	4.2	4452	3.4
**Start of delivery**	Spontaneous	216838	69.4	26006	72.9	54963	75.0	22129	77.2	1946	54.1	624	24.5	26318	70.0	84852	64.7
	Induction	66721	21.3	7342	20.6	15513	21.2	6337	22.1	815	22.7	148	5.8	11279	30.0	25287	19.3
	Cesarean section	28969	9.3	2316	6.5	2808	3.8	207	0.7	837	23.3	1770	69.6	0	0.0	21031	16.0
**Birthweight (grams)**	<4000	222509	71.2	26665	74.8	49843	68.0	19593	68.3	3327	92.5	2404	94.6	23258	61.9	97419	74.3
	4000–4499	68414	21.9	6928	19.4	17788	24.3	6956	24.3	222	6.2	118	4.6	10539	28.0	25863	19.7
	4500–4999	18621	6.0	1803	5.1	4911	6.7	1851	6.5	41	1.1	18	0.7	3245	8.6	6752	5.1
	≥5000	2984	1.0	268	0.8	742	1.0	273	1.0	8	0.2	2	0.1	555	1.5	1136	0.9
**Fetal sex** ^**b**^	Girl	154394	49.4	19241	54.0	37118	50.7	14021	48.9	1601	44.5	1184	46.6	16634	44.2	64595	49.2
	Boy	158114	50.6	16414	46.0	36163	49.3	14652	51.1	1997	55.5	1358	53.4	20962	55.8	66568	50.8
**Cesarean section (irrespective of start)**	No	237468	76.0	30622	85.9	65379	89.2	27751	96.8	1176	32.7	119	4.7	20995	55.8	91426	69.7
	Yes	75060	24.0	5042	14.1	7905	10.8	922	3.2	2422	67.3	2423	95.3	16602	44.2	39744	30.3
**Vacuum delivery**	No	275186	88.1	32333	90.7	66115	90.2	23458	81.8	3494	97.1	2537	99.8	22347	59.4	124902	95.2
	Yes	37342	11.9	3331	9.3	7169	9.8	5215	18.2	104	2.9	5	0.2	15250	40.6	6268	4.8
**Forceps delivery**	No	302559	96.8	34869	97.8	71598	97.7	26663	93.0	3547	98.6	2536	99.8	33700	89.6	129646	98.8
	Yes	9969	3.2	795	2.2	1686	2.3	2010	7.0	51	1.4	6	0.2	3897	10.4	1524	1.2
**Shoulder dystocia**	No	307711	98.5	35177	98.6	72058	98.3	27989	97.6	3586	99.7	2540	99.9	36558	97.2	129803	99.0
	Yes	4817	1.5	487	1.4	1226	1.7	684	2.4	12	0.3	2	0.1	1039	2.8	1367	1.0
**Episiotomy** ^**c**^	No	171611	77.9	19634	79.3	35045	75.5	13164	68.0	2218	96.5	2149	99.2	19842	64.7	79559	84.1
	Yes	48705	22.1	5114	20.7	11368	24.5	6202	32.0	80	3.5	18	0.8	10834	35.3	15089	15.9
**Epidural anasthesia**	No	217383	69.6	26376	74.0	54188	73.9	19013	66.3	3101	86.2	2268	89.2	13166	35.0	99271	75.7
	Yes	95145	30.4	9288	26.0	19096	26.1	9660	33.7	497	13.8	274	10.8	24431	65.0	31899	24.3
**Placenta defined as normal** ^**c**^	No	54383	24.7	18208	73.6	10490	22.6	3003	15.5	1050	45.7	714	32.9	6677	21.8	14241	15.0
	Yes	165933	75.3	6540	26.4	35923	77.4	16363	84.5	1248	54.3	1453	67.1	23999	78.2	80407	85.0
**Velamentous umbilical cord incertion** ^**c**^	No	215432	97.8	23625	95.5	45476	98.0	19002	98.1	2205	96.0	2052	94.7	30159	98.3	92913	98.2
	Yes	4884	2.2	1123	4.5	937	2.0	364	1.9	93	4.0	115	5.3	517	1.7	1735	1.8
**Marginal umbilical cord incertion** ^**c**^	No	206851	93.9	23027	93.0	43267	93.2	18090	93.4	2093	91.1	1949	89.9	28818	93.9	89607	94.7
	Yes	13465	6.1	1721	7.0	3146	6.8	1276	6.6	205	8.9	218	10.1	1858	6.1	5041	5.3

Incidences according to maternal, pregnancy and birth characteristics are given in Table 3.

^a^ 1999–2017, smoking status available in 163731 deliveries with PPH.

^b^ 18 newborns with unknown sex.

^c^ 1999–2017.

Young women were more often registered with PPH due to obstetric trauma, and women with PPH caused by dystocia and obstetric trauma were more often nulliparous.

Smoking was more common in PPH associated with placenta previa and placental abruption.

Diabetes mellitus was more common in PPH associated with dystocia (4.2%), while preeclampsia was more common in PPH associated with placental abruption (8.0%).

High birthweight was commonly found in PPH caused by dystocia, atony and obstetric trauma.

A history of first trimester bleeding was more common in women with PPH due to placenta previa (6.1%) and retained placenta (4.8%), while the opposite was the case for PPH caused by obstetric trauma (1.9%).

Women who experienced PPH due to retained placenta or atony were more likely delivering girls than boys, while those with PPH caused by dystocia, obstetric trauma and undefined bleeding cause were more often delivering boys.

Placenta was defined as “normal” (tic box) in most deliveries with PPH without defined cause, in PPH due to obstetric trauma, and due to dystocia (75–85%). The opposite was found for PPH caused by retained placenta, where 26% of the placentas were defined as normal.

Table 3 shows risks of PPH types according to maternal, pregnancy and birth characteristics. We selected deliveries that were induced or had spontaneous onset, and found that PPH due to placental abruption and placenta previa represented a small proportion (2%) of all PPH registrations, and these were therefore not included in Tables 3–5. The risk of PPH increased with maternal age, and the association was strongest for PPH due to dystocia, followed by retained placenta, undefined bleeding cause and obstetric trauma. The effects were attenuated by adjustment for year of birth, while including parity in the model strengthened the associations. The risk of PPH was highest in primiparas, regardless of PPH type, especially with PPH caused by dystocia and obstetric trauma. By including maternal age to the models these associations were strengthened.

**Table 3:** Risk of type specific postpartum hemorrhage (PPH >500ml) according to maternal, pregnancy and birth characteristics; singleton births, ≥22 weeks of gestation and spontaneous onset or induction of labor.

		Total	Retained placenta					Atony					Trauma or laceration					Dystocia					Undefined bleeding cause				
		(n)	(n)	%	aOR	95% CI		(n)	%	aOR	95% CI		(n)	%	aOR	95% CI		(n)	%	aOR	95% CI		(n)	%	aOR	95% CI	
**Maternal age (Years)**	<20	136032	841	0.6	0.55	0.51	0.59	2507	1.8	0.82	0.79	0.86	1049	0.8	0.55	0.51	0.59	801	0.6	0.33	0.31	0.36	3815	2.8	0.85	0.83	0.89
	20–24	652556	5540	0.8	0.74	0.72	0.77	14209	2.2	0.92	0.90	0.94	6035	0.9	0.73	0.70	0.75	5829	0.9	0.56	0.54	0.58	21328	3.3	0.93	0.91	0.94
	25–29	916125	10647	1.2	1	Ref		24536	2.7	1	Ref		10600	1.2	1	Ref		13315	1.5	1	Ref		36781	4.0	1	Ref	
	30–34	662257	10324	1.6	1.32	1.28	1.34	19722	3.0	1.03	1.01	1.05	7593	1.1	1.09	1.06	1.13	11806	1.8	1.42	1.38	1.46	31205	4.7	1.07	1.05	1.09
	35–39	260686	4932	1.9	1.62	1.56	1.68	8012	3.1	1.06	1.03	1.09	2751	1.1	1.16	1.10	1.22	4914	1.9	1.79	1.73	1.86	13969	5.4	1.17	1.14	1.20
	40–44	46563	929	2.0	1.75	1.62	1.89	1395	3.0	1.09	1.03	1.15	410	0.9	1.14	1.02	1.28	884	1.9	2.04	1.90	2.20	2819	6.1	1.34	1.29	1.40
	≥45	2198	43	2.0	1.97	1.43	2.70	76	3.5	1.40	1.11	1.76	26	1.2	1.80	1.18	2.74	48	2.2	2.50	1.83	3.41	164	7.5	1.79	1.53	2.09
**Parity**	0	1124388	15284	1.4	1	Ref		33966	3.0	1	Ref		18609	1.7	1	Ref		28690	2.6	1	Ref		50806	4.5	1	Ref	
	1	935991	11389	1.2	0.77	0.75	0.79	23415	2.5	0.78	0.77	0.79	7409	0.8	0.42	0.41	0.44	6838	0.7	0.22	0.22	0.23	37766	4.0	0.86	0.85	0.88
	2	423535	4700	1.1	0.64	0.62	0.67	9225	2.2	0.66	0.65	0.68	1872	0.4	0.22	0.21	0.24	1511	0.4	0.10	0.09	0.11	14758	3.5	0.74	0.73	0.76
	3	128264	1273	1.0	0.55	0.51	0.58	2564	2.0	0.59	0.57	0.62	407	0.3	0.16	0.15	0.18	372	0.3	0.08	0.07	0.09	4305	3.4	0.71	0.69	0.74
	4	46957	412	0.9	0.48	0.43	0.53	868	1.8	0.52	0.57	0.62	131	0.3	0.14	0.11	0.16	135	0.3	0.08	0.06	0.09	1721	3.7	0.77	0.73	0.81
	≥5	17282	198	1.1	0.56	0.48	0.66	419	2.4	0.66	0.59	0.73	36	0.2	0.09	0.06	0.13	51	0.3	0.07	0.05	0.09	725	4.2	0.82	0.75	0.89
**Previous 1st trimester spontaneous abortion** ^**a**^	No	818952	17401	2.1	1	Ref		35221	4.3	1	Ref		16047	2.0	1	Ref		25314	3.1	1	Ref		60431	7.4	1	Ref	
	Yes	188824	5032	2.7	1.24	1.19	1.28	8437	4.5	1.09	1.07	1.12	3118	1.7	0.97	0.93	1.02	5362	2.8	1.07	1.04	1.11	13960	7.4	0.99	0.97	1.01
**1st trimester bleeding**	No	2628264	31692	1.2	1	Ref		68462	2.6	1	Ref		27927	1.1	1	Ref		36477	1.4	1	Ref		107109	4.1	1	Ref	
	Yes	48153	1564	3.2	2.10	1.99	2.22	1995	4.1	1.28	1.22	1.34	537	1.1	0.83	0.75	0.91	1120	2.3	1.10	1.03	1.17	2972	6.2	1.16	1.12	1.21
**Previous cesarean section**	No	2560531	31040	1.2	1	Ref		66385	2.6	1	Ref		25783	1.0	1	Ref		33216	1.3	1	Ref		101631	4.0	1	Ref	
	Yes	115886	2216	1.9	1.39	1.33	1.46	4072	3.5	1.27	1.22	1.31	2681	2.3	3.41	3.25	3.58	4381	3.8	6.08	5.82	6.35	8450	7.3	1.55	1.52	1.59
**Start of labor**	Spontaneous	2247799	25951	1.2	1	Ref		54953	2.4	1	Ref		22128	1.0	1	Ref		26318	1.2	1	Ref		84815	3.8	1	Ref	
	Induction	428618	7305	1.7	1.36	1.32	1.39	15504	3.6	1.41	1.38	1.43	6336	1.5	1.31	1.27	1.35	11279	2.6	1.87	1.82	1.91	25266	5.9	1.45	1.43	1.47
**Birthweight (grams)**	<2500	91074	1443	1.6	1.39	1.32	1.47	1073	1.2	0.42	0.39	0.45	245	0.3	0.22	0.19	0.25	191	0.2	0.12	0.11	0.14	3302	3.6	0.84	0.81	0.87
	2500–2999	271661	2829	1.0	0.85	0.82	0.89	3579	1.3	0.45	0.44	0.47	1343	0.5	0.38	0.36	0.41	1178	0.4	0.23	0.22	0.25	7915	2.9	0.64	0.63	0.66
	3000–3499	845937	8638	1.0	0.83	0.81	0.86	16106	1.9	0.66	0.65	0.68	6710	0.8	0.64	0.62	0.67	7351	0.9	0.50	0.49	0.52	27869	3.3	0.75	0.73	0.76
	3500–3999	957654	11706	1.2	1	Ref		26825	2.8	1	Ref		11114	1.2	1	Ref		14538	1.5	1	Ref		41202	4.3	1	Ref	
	4000–4499	415535	6669	1.6	1.32	1.28	1.36	17389	4.2	1.56	1.52	1.59	6934	1.7	1.69	1.63	1.75	10539	2.5	1.98	1.92	2.03	22974	5.5	1.32	1.29	1.34
	4500–4999	83839	1727	2.1	1.71	1.62	1.80	4784	5.7	2.22	2.15	2.29	1845	2.2	2.59	2.46	2.73	3245	3.9	3.61	3.46	3.76	5886	7.0	1.75	1.70	1.80
	≥5000	10717	244	2.3	1.93	1.70	2.20	701	6.5	2.73	2.53	2.94	273	2.5	3.43	3.03	3.89	555	5.2	5.78	5.26	6.35	933	8.7	2.29	2.14	2.45
**Fetal sex** ^**a**^	Girl	1300887	18026	1.4	1	Ref		35733	2.7	1	Ref		13901	1.1	1	Ref		16634	1.3	1	Ref		54477	4.2	1	Ref	
	Boy	1375459	15225	1.1	0.80	0.78	0.82	34722	2.5	0.92	0.90	0.93	14563	1.1	0.99	0.97	1.01	20962	1.5	1.18	1.16	1.21	55601	4.0	0.96	0.95	0.97
**Placental weight (grams)** ^**b**^	<500	92475	3759	4.1	2.03	1.95	2.11	2361	2.6	0.72	0.68	0.75	1223	1.3	0.74	0.80	0.75	1175	1.3	0.52	0.48	0.55	5623	6.1	0.89	0.87	0.92
	500–699	488935	9892	2.0	1	Ref		17118	3.5	1	Ref		8319	1.7	1	Ref		10732	2.2	1	Ref		31685	6.5	1	Ref	
	700–899	328966	6216	1.9	0.93	0.90	0.96	17552	5.3	1.55	1.51	1.59	7201	2.2	1.42	1.37	1.48	13355	4.1	2.07	2.02	2.12	27784	8.4	1.37	1.34	1.39
	900–1099	64314	1451	2.3	1.12	1.06	1.19	4995	7.8	2.32	2.24	2.40	1837	2.9	2.01	1.89	2.13	4175	6.5	3.74	3.60	3.89	6751	10.5	1.78	1.73	1.83
	≥1100	9934	270	2.7	1.37	1.21	1.54	933	9.4	2.85	2.65	3.06	336	3.4	2.59	2.28	2.94	746	7.5	4.62	4.26	5.00	1245	12.5	2.20	2.07	2.34
**Velamentous cord insertion** ^**c**^	No	992869	21427	2.2	1	Ref		42830	4.3	1	Ref		18809	1.9	1	Ref		30159	3.0	1	Ref		73055	7.4	1	Ref	
	Yes	14907	1006	6.7	3.00	2.79	3.21	828	5.6	1.26	1.17	1.36	356	2.4	1.21	1.07	1.37	517	3.5	0.99	0.90	1.09	1336	9.0	1.24	1.17	1.31
**Marginal umbilical cord insertion** ^**c**^	No	953165	20948	2.2	1	Ref		40821	4.3	1	Ref		17907	1.9	1	Ref		28818	3.0	1	Ref		70568	7.4	1	Ref	
	Yes	54611	1485	2.7	1.26	1.19	1.33	2837	5.2	1.24	1.19	1.29	1258	2.3	1.25	1.17	1.33	1858	3.4	1.12	1.06	1.18	3823	7.0	1.03	0.90	1.06

CI confidence interval, aOR: OR adjusted for maternal age, parity and period (1967–1977, 1978–1987, 1988–1997, 1998–2007 and 2008–2017).

^a^ 71 newborns with unknown sex. The negative association with PPH due to trauma or laceration disappeared after adjusting for unmeasured confounder or stratification by preterm/term delivery.

^b^ 1999–2017, 23152 without placental weight.

^c^ 1999–2017.

**Table 4:** Risk of type specific postpartum hemorrhage (PPH>500ml) in the second delivery according to PPH types in the first delivery and pregnancy- and birth characteristic; singleton births, ≥22 weeks of gestation and spontaneous onset or induction of labor.

			Type of PPH in second delivery																								
Type of PPH in first delivery and pregnancy/birth related exposures		Total	Retained placenta					Atony					Trauma or laceration					Dystocia					Undefined bleeding cause				
		(n)	(n)	%	aOR	95% CI		(n)	%	aOR	95% CI		(n)	%	aOR	95% CI		(n)	%	aOR	95% CI		(n)	%	aOR	95% CI	
**Retained placenta**	No	785902	9107	1.2	1	Ref		19178	2.4	1	Ref		5795	0.7	1	Ref		4811	0.6	1	Ref		30674	3.9	1	Ref	
	Yes	8788	734	8.4	5.90	5.45	6.39	729	8.3	2.93	2.71	3.17	155	1.8	1.77	1.50	2.08	132	1.5	1.56	1.31	1.86	841	9.6	1.94	1.80	2.09
**Atony**	No	773349	8950	1.2	1	Ref		17745	2.3	1	Ref		5517	0.7	1	Ref		4612	0.6	1	Ref		29601	3.8	1	Ref	
	Yes	21341	891	4.2	2.91	2.71	3.13	2162	10.1	4.00	3.82	4.20	433	2.0	2.15	1.95	2.37	331	1.6	1.77	1.58	1.98	1914	9.0	1.90	1.81	1.99
**Trauma or laceration**	No	783198	9475	1.2	1	Ref		19031	2.4	1	Ref		5513	0.7	1	Ref		4855	0.6	1	Ref		30415	3.9	1	Ref	
	Yes	11492	366	3.2	2.01	1.81	2.24	876	7.6	2.65	2.47	2.85	437	3.8	3.86	3.49	4.27	88	0.8	0.77	0.63	0.96	1100	9.6	1.89	1.77	2.02
**Dystocia**	No	780869	9251	1.2	1	Ref		18800	2.4	1	Ref		5498	0.7	1	Ref		4080	0.5	1	Ref		29915	3.8	1	Ref	
	Yes	13821	590	4.3	2.44	2.24	2.66	1107	8.0	2.58	2.42	2.75	452	3.3	2.98	2.70	3.29	863	6.2	6.81	6.31	7.36	1600	11.6	2.09	1.98	2.21
**Undefined bleeding cause**	No	764798	9082	1.2	1	Ref		18281	2.4	1	Ref		5419	0.7	1	Ref		4501	0.6	1	Ref		28367	3.7	1	Ref	
	Yes	29892	759	2.5	1.64	1.52	1.77	1626	5.4	1.90	1.80	2.00	531	1.8	1.80	1.64	1.97	442	1.5	1.62	1.46	1.79	3148	10.5	2.21	2.12	2.30
**Inter-delivery interval (year)**	<1	3433	56	1.6	2.00	1.53	2.62	64	1.9	0.90	0.70	1.15	10	0.3	0.57	0.31	1.07	12	0.3	1.14	0.64	2.02	118	3.4	1.01	0.84	1.22
	1 to <2	151853	1815	1.2	1.08	1.01	1.14	3723	2.5	0.99	0.95	1.03	1001	0.7	0.92	0.86	1.00	809	0.5	1.00	0.91	1.09	5905	3.9	0.98	0.85	1.02
	2 to <3	239848	2924	1.2	1	Ref		6255	2.6	1	Ref		1906	0.8	1	Ref		1507	0.6	1	Ref		9898	4.1	1	Ref	
	3 to <4	166293	2118	1.3	1.08	1.02	1.15	4208	2.5	1.01	0.97	1.05	1319	0.8	1.05	0.98	1.12	968	0.6	0.98	0.91	1.07	6578	4.0	1.01	0.98	1.05
	4 to <5	88863	1046	1.2	1.02	0.95	1.10	2128	2.4	0.97	0.92	1.02	627	0.7	0.96	0.88	1.05	557	0.6	1.10	1.00	1.21	3364	3.8	1.01	0.97	1.05
	≥5	144400	1882	1.3	1.00	0.94	1.06	3529	2.4	0.93	0.89	0.97	1087	0.8	0.95	0.88	1.02	1090	0.8	1.10	1.02	1.19	5652	3.9	1.00	0.96	1.03
**Change of father** ^**a**^	No	713133	8704	1.2	1	Ref		17912	2.5	1	Ref		5351	0.8	1	Ref		4303	0.6	1	Ref		28160	3.9	1	Ref	
	Yes	69371	981	1.4	1.00	0.94	1.07	1676	2.4	0.86	0.81	0.90	515	0.7	0.88	0.80	0.96	516	0.7	1.02	0.93	1.12	2808	4.0	0.93	0.90	0.97
**Previous cesarean** ^**b**^	No	743587	8893	1.2	1	Ref		18127	2.4	1	Ref		4518	0.6	1	Ref		2270	0.3	1	Ref		27723	3.7	1	Ref	
	Yes	65619	1312	2.0	1.35	1.28	1.44	2368	3.6	1.28	1.23	1.34	1929	2.9	4.00	3.79	4.23	3368	5.1	13.16	12.46	13.89	5125	7.8	1.84	1.78	1.90

CI confidence interval, aOR OR adjusted for maternal age, parity and period (1967–1977, 1978–1987, 1988–1997, 1998–2007 and 2008–2017).

^a^ 12186 births with unknown father in 1st or 2nd delivery.

^b^ First deliveries starting with cesarean section included.

**Table 5:** Risk of postpartum hemorrhage (PPH>500ml) due to dystocia in second deliveries without previous cesarean section (CS) (reference), second deliveries with previous CS, and first deliveries; singleton births, ≥22 weeks of gestation and spontaneous onset or induction of labor.

	Total	Dystocia related PPH				
Groups	(n)	(n)	%	aOR	95% CI	
**2nd delivery without previous CS**	864751	3146	0.4	1	Ref	
**2nd delivery with previous CS**	71240	3692	5.2	18.85	17.84	19.92
**1st delivery**	1124388	28690	2.6	9.10	8.72	9.48

CI confidence interval, aOR OR adjusted for maternal age and period (1967–1977, 1978–1987, 1988–1997, 1998–2007 and 2008–2017).

First trimester bleeding was associated with a doubled risk of PPH due to retained placenta and had weaker association with PPH due to atony and without defined cause.

The risks of PPH types included in Table 3 increased with birthweight, especially PPH due to dystocia, obstetric trauma and atony. Including parity, maternal age and year of delivery in the models strengthened the associations, mainly for PPH due to dystocia and obstetric trauma. In term but not preterm deliveries, low birthweight (<2500g) was associated with PPH due to retained placenta and/or membranes.

Exploring the effect of fetal sex on the PPH types, we found that the risk of PPH was lower if the newborn was a boy. This association was strongest for PPH due to retained placenta (aOR: 0.80, 95% CI 0.78–0.82), followed by atony (aOR 0.92, 95% CI: 0.90–0.93) and undefined cause of PPH (aOR 0.96, 95% CI: 0.95–0.97). These associations were similar in strata of birthweight (<2500g, 2500–2999g, 3000–3499g, 3500–4000g, 4000–4499g, 4500–4999 g, ≥5000g). Adjusted OR for PPH due to obstetric trauma was also lower for deliveries of a boy, but this effect was only significant in weight groups between 3000 and 4499g. However, if the newborn was a boy, there was increased risk of PPH due to dystocia, but this association disappeared after stratification according to birthweight.

The association between placenta weight categories and the specific causes of PPH generally showed a pattern like that of birthweight.

Velamentous and marginal umbilical placental cord insertion were strongest associated with PPH due to retained placenta. This effect was significantly stronger for velamentous- (aOR: 3.1, 95% CI: 2.9–3.4) than marginal cord insertion (aOR: 1.3, 95% CI: 1.2–1.3).

Table 4 shows the risk of PPH types in the second delivery (except for PPH caused by placental abruption and placenta previa) according to PPH types in the first delivery and pregnancy and birth related factors.

The risk of recurrent PPH was strongest for the same type. PPH associated with dystocia had highest risk of recurrence (aOR: 6.8, 95% CI: 6.3–7.4), followed by PPH due to retained placenta and/or membranes (aOR: 5.9, 95% CI: 5.5–6.4), atony (aOR: 4.0, 95% CI: 3.8–4.2) and obstetric trauma (aOR: 3.9, 95% CI: 3.5–4.3), while PPH of undefined cause had lowest risk of recurrence (aOR: 2.2, 95% CI: 2.1–2.3) (Table 4).

Exploring effects of pregnancy related factors on the PPH types in the second delivery, we found that inter-delivery interval had no significant effect on the PPH risk in second delivery, except for PPH due to retained placenta where a short inter-delivery interval (less than one year) was associated with a doubled risk (aOR: 2.0, 95% CI: 1.5–2.6).

Change of father slightly decreased ORs of PPH due to obstetric trauma, atony and undefined bleeding cause. Additional adjustment for inter-delivery interval did not influence the associations.

A previous cesarean delivery was associated with a marked increased risk of PPH due to dystocia, (aOR of 13.2, 95% CI: 12.5–13.9), and a weaker association with PPH caused by obstetric trauma (aOR: 4.0, 95% CI: 3.8–4.2), undefined PPH, retained placenta and atony (aORs between 1.3 and 1.8). In additional analyses we compared risks of PPH associated with dystocia in three groups: second deliveries without previous cesarean section (reference), second deliveries with previous cesarean section, and first deliveries (Table 5). We found that the risk of PPH due to dystocia was higher in women with a previous cesarean (vaginal primiparas) than in primiparas.

Our sensitivity analyses (S1 File) indicated that the associations described in Tables 3 and 4 persisted after adjusting for potential unmeasured confounders, and that false positive associations due to multiple testing were not present.

## Discussion

### Main findings

We found that maternal, fetal and obstetric characteristics had differential effects on the types of PPH. The risk of recurrence differed considerably between the PPH types; the strongest recurrence risks were found for PPH caused by dystocia, retained placenta and atony. PPH due to retained placenta was most prone to develop into severe PPH.

### Strengths and limitations

A main strength of the study was the long study period with mandatory registration of all births in the country, and with almost complete record linkage, which made it possible to do comprehensive sub-analyses. We also consider it a strength that it has been possible to classify clinically relevant causes of PPH since the inception of the registry. The population-based design and prospective collection of data attenuate selection and recall bias. Ethically, this is the study design of choice as we investigate a potentially life-threatening outcome [28]. Furthermore, the PPH-variable has been validated and found to be of adequate quality for epidemiological studies [29]. The robustness of our results for potentially unknown confounding variables, assessed in the sensitivity analyses, is reassuring.

The introduction of activity-based financing and update of the MBRN registration form in 1999 may have improved the registration and contributed to the increased occurrence of PPH without specified cause after 1999, representing 29.2 percent of all registered PPH cases in the total study period.

It is possible that misclassification between types of PPH occurs, for example between retained placenta and atony. We expect that such misclassification to be non-differential and would therefore not affect the ORs. Coexistence of more than one PPH type in a delivery, for example atony and obstetric trauma caused by macrosomia is plausible, and there was no upper limit for registration of types of PPH in each delivery.

### Previous studies

#### International variation and demographic factors

In contrast to the situation worldwide, the maternal mortality rate of PPH in Norway is low [1,30], which may limit the generalizability of our results. However, in other settings we assume that proportions of severe bleeding in the different types of PPH may show similar pattern.

There are considerable differences in the reported proportions of PPH types in the literature, especially for PPH caused by atony and retained placenta. Bateman et al. [12] and Widmer et al. [2] reported that 79% and 62% of all registered PPH cases (>500ml and refractory PPH, respectively) were accounted for by atony, which is in contrast with our findings (23% PPH due to atony) (Table 1). Our result is more in line with the 41% due to atony in a Swedish study (>1000ml) [3]. The proportion of PPH due to retained placenta in our study (11.4%) is in line with other studies [5,12]. Oberg et al. reported that 33.5% of PPH cases were due to retained placenta, which is comparable to our results in severe PPH (25.7% due to retained placenta) [3]. Additionally, a Danish study found higher proportions of retained placenta in severe PPH, compared to milder PPH [5], and a Turkish study found retained placenta to be associated with severe PPH [31]. These results agree with our results that PPH due to retained placenta most often caused severe PPH (Table 1).

These inter-study variations may be caused by differences in code availability or definitions of excessive bleeding, although it cannot be ruled out that variations of population genetic and/or environmental properties, or medical culture, may also play a role.

Our results confirm that maternal age was associated with all types of PPH (with the strongest association for PPH caused by dystocia). The effect of maternal age on PPH caused by atony are in line with existing knowledge [2,4,6,7,32]. Studies on associations between maternal age and other types of PPH are scarce, but an association with retained placenta in general has been reported [32]. Parity had strongest effect on PPH due to dystocia; 76% of the cases were primiparas, which agrees with the higher risk of dystocia in nulliparas [33].

As dystocia may result in uterine fatigue and atony, PPH due to dystocia may have been classified as atony in studies where dystocia is not recorded in the databases. This may, at least in part, explain the very high proportion of PPH due to atony found in some studies [12].

#### Pregnancy-related factors

We found a slightly reduced risk of recurrent PPH (caused by obstetric trauma and atony and undefined bleeding cause) in mothers who had changed partner, also after adjusting for inter-delivery interval. This fits with our previous findings of a weak but significant paternal effect on recurrent PPH [10]. In the present study there was a significantly increased risk of PPH due to retained placenta when the inter-delivery interval was short (less than one year). This contrast findings regarding PPH in general, where inter-delivery interval had a negligible effect on recurrence [10].

The association of first trimester bleeding and PPH caused by retained placenta is consistent with results from previous studies that retained placenta [34] and PPH in general [35] are associated with threatened abortion.

#### Obstetric history (including recurrence)

Recurrence risk of PPH due to retained placenta [3,5,8,11], atony and laceration [3], and increased duration and pushing time of the second stage of labor have been associated with PPH [36], which is in line with our results. However, we found that PPH caused by dystocia was the PPH type most prone to recur, which to our knowledge has not been reported before.

A history of cesarean section has been linked to risk of retained placenta in general [6,13,16] and atonic PPH [12], but not consistently [8,37]. In our population women with a previous cesarean carried increased risk of all causes of PPH, but the strongest association was found with dystocia PPH (Table 4).

#### Complications related to the fetus, placenta, membranes and umbilical cord

The finding that birthweight has a strong association to PPH (Table 3) is in line with previous findings [10,38,39]. However, a new finding was that the strength of associations markedly varied with type of PPH, and that birthweight had the strongest association with PPH due to dystocia.

Sex differences in properties of placenta, umbilical cord and birthweight are well known [40–44]. We found a strong effect of fetal sex on most types of PPH and especially for PPH caused by retained placenta. This was a new finding and is consistent with previous findings that delivery of girls carries higher risk of retained placenta in general [8,13] and PPH due to atony [45].

As expected, PPH without specific cause was dominated by mild cases. Its low risk of recurrence is in line with the concept that a mild phenotype of a polygenic trait or disease is generally less prone to recur than a severe phenotype [46]. This suggests that most of these cases were correctly assigned to the group.

### Interpretation

Risk factors for dystocia, with and without PPH, have previously been reported [36,47–49], but the strong recurrence risk of PPH due to dystocia has to our knowledge not been studied before. As dystocia may be an indication for operative delivery, this may result in PPH due to trauma to the birth canal. The recurrence risk of PPH due to dystocia may be caused by sustained or recurrent factors associated with PPH or indicative of operative delivery, such as tendency to deliver large babies and fetopelvic disproportion. Further, dystocia may lead to atonic PPH through exhausting workload on the uterus without adequate progression of labor.

It is reasonable to assume that the placenta accreta spectrum constitutes some of the cases of severe PPH in the retained placenta group. However, we do not have exact information on the occurrence of placenta accreta spectrum in our population, and this was beyond the scope of our study. Another possible explanation for the higher occurrence of severe PPH among women with PPH due to retained placenta is the lack of effective initial medical treatment, along with the need of surgical intervention which may be delayed. In contrast, atony often is sufficiently treated with medications.

A previous cesarean was strongly associated with PPH due to dystocia in the second delivery, and we also found associations with PPH due to obstetric trauma, retained placenta and atony. The risk of PPH due to dystocia was higher than in nulliparas. A possible explanation for the association of previous cesarean section with PPH due to dystocia may be ineffective labor contractions due to the uterine scar, and that no previous vaginal delivery may mimic a primipara, with increased risk of delayed progression in labor and exhaustion of uterine contractility. One may speculate that the association of previous cesarean section with PPH due to retained placenta is associated with an early stage of abnormally invasive placenta, consistent with the increased risk of abnormally invasive placenta in women with previous cesarean section [50].

We found that birthweight was associated with all types of PPH, but especially PPH due to dystocia, birth canal lacerations and uterine atony. This was expected, as macrosomia is associated with PPH through distention of the uterus and large utero-placental wound surface [2,10,14,51]. In addition, macrosomia may increase tension on maternal tissue during labor leading to increased risk of obstetric trauma [52]. Another explanatory mechanism is that fetal macrosomia, dystocia and atony may be indications for operative vaginal delivery and result in surgical bleeding.

A possible explanation for the reduced risk of PPH due to retained placenta if the newborn was a boy (Table 3) may be the more inadequate transformation of the uterine spiral arteries in pregnancies with male fetus [53–55]. This agrees with the fetal sex preponderance in complications of the placenta, like placental abruption [45] and preeclampsia [56], although not consistently for the latter [45].

To increase the relevance for clinical practice we analyzed deliveries with spontaneous onset or induction of labor, thus excluding cesarean sections before the onset of labor. Deliveries with PPH due to placenta previa or placental abruption are underrepresented in our material (only 2% of PPH cases) since they primarily are delivered by cesarean section before labor and were therefore not included in the main analyses.

The substantial variation of reported incidence of causes of PPH among populations call for initiatives to unite the international definitions and improve the understanding of PPH pathophysiological mechanism.

We have already addressed the need of alertness when a delivering woman or her relatives has experienced PPH [9,10]. Based on our present results, we encourage special attention concerning PPH due to retention of placenta or membranes, as its recurrence risk is high, and that a retained placenta carried the highest risk of severe PPH.

PPH due to retention of placenta or membranes was related to velamentous and marginal umbilical cord insertion in a dose-response-pattern with strongest association to velamentous insertion. Both conditions are possible to diagnose by ultrasonography during pregnancy [57]. Thus, prenatal identification of an abnormal cord insertion may serve to alert clinicians and enhance their preparedness.

We found a strong association between previous cesarean section and PPH due to dystocia, and that it was likely to recur from the first to the second delivery. Dystocia is widely ignored as a cause of PPH in the literature, but our study indicates that a history of PPH due to dystocia should be included in risk assessment for PPH.

## Conclusions

In this large population-based study we found that maternal, fetal and obstetric characteristics had differential effects on types of PPH. Recurrence differed considerably between PPH types. Retained placenta was most frequently registered with severe PPH, and showed strongest effect of sex; delivery of a boy was associated with lower risk of PPH. Previous cesarean increased the risk of PPH due to dystocia.

Our research adds to the understanding of recurrence risk of PPH and suggests that PPH can be inherited. In future studies genetic influence on specific types of PPH needs to be disentangled from environmental influence.

## Supporting information

S1 File Statistical analysis. (DOCX)

## Abbreviations

aOR: adjusted odds ratio
CI: confidence interval
MBRN: Medical Birth Registry of Norway
OR: odds ratio
PPH: postpartum hemorrhage
