# Supplementary material for: Risk factors and recurrence of cause-specific postpartum hemorrhage: A population-based study
Source: PLoS One. 2022 Oct 14;17(10):e0275879. doi: 10.1371/journal.pone.0275879 (PMC9565392; doi:10.1371/journal.pone.0275879)
Supplement: S1 File — (DOCX) [file pone.0275879.s001.docx]

**S1 Supporting information (Statistical analysis)**

**Independent variables**

Independent variables were variables related to demographic characteristics, obstetric history, pregnancy and fetal complications, and placental/membranes/umbilical cord characteristics. Independent variables also included a history of previous postpartum hemorrhage (PPH) type in the first delivery, inter-delivery interval, change of father between pregnancies, first or second delivery bleeding before 13 weeks of gestation, and previous cesarean section.

These analyses included the following possible confounding factors: maternal age (<20 years, 20–24 years, 25–29 years, 30–34 years, 35–39 years or ≥40 years), parity (0, 1, 2, 3, 4 or ≥5 ), inter-delivery interval (<1 year, 1 to <2 years, 2 to <3 years, 3 to <4 years, 4 to <5 years or ≥5 years), marital status (married/registered partner, cohabitating, not married/alone, divorced/separated/widow, not defined), mother’s country of birth (Norway or eight WHO regions) (1) (A) high-income countries, (B) Central Europe, Eastern Europe and Central Asia, (C) sub-Saharan Africa, (D) North Africa and Middle East, (E) South Asia, (F) Southeast Asia, East Asia and Oceania, (G) Latin America and Caribbean or (H) unknown or stateless], level of education (available until 2013) (<8 years, 8–10 years, 11–12 years, 13–17 years, ≥18 years or no information), and the period of birth divided into five groups with approximately equal durations (1967–1977, 1978–1987, 1988–1997, 1998–2007 and 2008–2017).

**Statistical analysis**

We used multilevel logistic regression analyses to calculate odds ratios (ORs) with 95% confidence intervals (CIs) for PPH types in the second delivery as outcomes, and variables related to demographic characteristics, obstetrical history, pregnancy and fetal complications, and placental/ membranes/ umbilical cord characteristics as exposures. We also calculated ORs for PPH types in the actual birth as outcomes and a history of PPH type as exposure variables.

We accounted for the hierarchical nature of the family data by performing multilevel regression analyses in which the data were sorted into different levels in analyses including one or more births of the same parent: current delivery (level 1) and parent (level 2). Possible confounding variables were included if they were associated with PPH in the current delivery and the exposure.

We performed sensitivity analyses to assess if the associations studied persisted after adjusting for unmeasured confounders (2) and to indicate potentially false positive associations caused by multiple testing (3). We implemented a Markov Chain Monte Carlo simulation (4) in which we entered the regression models and a prior assumption. The prior assumption was that adding an influential, unmeasured confounder to known confounder(s) would zero out the association (null hypothesis), decreasing the regression coefficient (β; standard deviation) for the main exposure variable (PPH) to 0; 0.05, corresponding to an OR of 1 with a 95% CI of 0.9–1.1. In order to simulate confounding, we entered a simple regression model (fixed effect: β_0_ + β_1_ PPH (0 or 1), where β_0_ and β_1_ are constants), and calculated the effects (ORs of PPH) before and after including the prior assumption. The statistical analyses were performed using SPSS (version 25) and MLwiN (version 3.05).

**References**

1. World Health Organization: Intrapartum care for a positive childbirth experience. Geneva: World Health Organization; 2018. Licence: CC BY-NC-SA 3.0 IGO. [Available from: <https://www.who.int/reproductivehealth/publications/intrapartum-care-guidelines/en/>].

2. Greenland S. Bayesian perspectives for epidemiologic research: III. Bias analysis via missing-data methods. International journal of epidemiology. 2009;38(6):1662-73.

3. Shikano S. Bayesian estimation of regression models. The SAGE Handbook of Regression Analysis and Causal Inference. p. 31-54.

4. Browne WJ. MCMC Estimation in MLwiN, v3.03: Centre for Multilevel Modelling, University of Bristol; 2019.
